# Co-Transcriptional RNA Processing in Plants: Exploring from the Perspective of Polyadenylation

**DOI:** 10.3390/ijms22073300

**Published:** 2021-03-24

**Authors:** Jing Yang, Ying Cao, Ligeng Ma

**Affiliations:** College of Life Sciences, Capital Normal University, Beijing 100048, China; jingyang_cell@163.com (J.Y.); cy005@163.com (Y.C.)

**Keywords:** polyadenylation, RNA processing, transcription, coupling regulation, liquid–liquid phase separation (LLPS), gene expression, plant

## Abstract

Most protein-coding genes in eukaryotes possess at least two poly(A) sites, and alternative polyadenylation is considered a contributing factor to transcriptomic and proteomic diversity. Following transcription, a nascent RNA usually undergoes capping, splicing, cleavage, and polyadenylation, resulting in a mature messenger RNA (mRNA); however, increasing evidence suggests that transcription and RNA processing are coupled. Plants, which must produce rapid responses to environmental changes because of their limited mobility, exhibit such coupling. In this review, we summarize recent advances in our understanding of the coupling of transcription with RNA processing in plants, and we describe the possible spatial environment and important proteins involved. Moreover, we describe how liquid–liquid phase separation, mediated by the C-terminal domain of RNA polymerase II and RNA processing factors with intrinsically disordered regions, enables efficient co-transcriptional mRNA processing in plants.

## 1. Introduction

Following their transcription from genomic DNA, most nascent RNAs in eukaryotes are processed via multiple steps, including capping, splicing, polyadenylation, and chemical modification, resulting in mature functional messenger RNAs (mRNAs) [1]. Many studies have focused on the mechanisms of splicing and chemical modification, but relatively few studies have considered polyadenylation. Polyadenylation is an important step in gene expression; it affects transcript localization [2], mRNA stability [3,4], translation efficiency [5,6], the nuclear export of mRNAs [7,8], and other essential biochemical processes [9]. The process of polyadenylation includes cleavage at a poly(A) site and the addition of a poly(A) tract [4]. Polyadenylation at different sites of the same gene is called alternative polyadenylation (APA), and it contributes greatly to the complexity of gene expression [1]. APA is widespread among eukaryotes; more than 60% of plant protein-coding genes express alternatively polyadenylated isoforms, and more than 70% of *Arabidopsis* genes have at least two poly(A) sites [10,11].

In this review, we briefly introduce polyadenylation in plants, and we describe recent advances in our understanding of the regulatory events that couple polyadenylation with transcription, splicing, and the methylation of adenosine at the N6 position (m^6^A). We then propose a possible spatial environment for this coupling and describe important proteins that are part of the coupling machinery. Finally, we outline a possible pathway whereby such coupling helps regulate gene expression in plants.

## 2. A Brief Overview of Polyadenylation in Plants

As sessile organisms, plants have evolved specific strategies for responding to environmental changes, and the polyadenylation of pre-mRNAs, which is an important part of gene regulation, plays a key role in plant development and stress responses.

### 2.1. The Core Polyadenylation Apparatus in Plants

The polyadenylation of most eukaryotic mRNAs involves two steps: cleavage at the appropriate site at the 3′ end of the molecule and the addition of a poly(A) tail. The machinery includes about 85 proteins [12] and multiple sequence elements in the nascent RNA [1]. According to studies of mammalian and yeast cells, the core 3′ cleavage and polyadenylation apparatus includes four complexes: cleavage and polyadenylation specificity factor (CPSF), cleavage stimulation factor (CstF), cleavage factor I (CFI), and cleavage factor II (CFII). These complexes cooperate with poly(A) polymerase (PAP) and various RNA-binding proteins (RBPs) to cleave and polyadenylate nascent RNAs [12,13]. These cleavage and polyadenylation factors work in close collaboration with each other via the recognition of specific sequence elements along the nascent RNA, such as the highly conserved hexamer AAUAAA and its 11 single-nucleotide variants; in humans, up to 82.5% of pre-mRNAs possess this canonical polyadenylation signal (PAS) [13,14]. Nearly all of these core cleavage and polyadenylation factors have homologues in plants (Table 1); however, the hexamer AAUAAA and its 11 single-nucleotide variants are seen in only 8–12% of the transcripts in *Arabidopsis thaliana* (L.) Heynh and rice [14]. How plants are able to cleave and polyadenylate pre-mRNAs precisely without the use of highly conserved PASs is an interesting question.

### 2.2. Polyadenylation Is Crucial for Plant Development and Stress Responses

A variety of growth defects have been reported in several polyadenylation factor mutants. For example, *cstf77-2*, a T-DNA insertion mutant of *cleavage stimulation factor 77*, exhibits curled cotyledons, short roots, a long hypocotyl, and a reduced cell number in the root meristem at the seedling stage, and late flowering and dwarfism in adult plants [28]. Meanwhile, a decreased number of emerged lateral roots, disorganized quiescent center, and reduced stem cell niche were reported in *fip1-2* (affecting factor interacting with poly[A] polymerase 1 (Fip1), a core component of the CPSF complex) [29]. Other defects have been detected, like reduced female gametophyte transmission in a mutant of CLPS3 (an ortholog of the human polyadenylation factor CLP1) [25] and altered flowering time in a poly(A) polymerase mutant [30]. Further, homozygous mutations affecting polyadenylation factors have been shown to be lethal [31]. Together, these observations show the indispensable role of polyadenylation in plant development.

Moreover, genome-wide induction of the 3′-untranslated region (UTR) extensions has been observed under conditions of dehydration [32], and increased usage of proximal poly(A) sites (within the 5′-UTR, introns, and protein-coding regions) was observed in response to hypoxia in plants [33]. Global changes in APA have also been detected under conditions of biotic stress (e.g., bacterial blight, rice blast, and rice stripe virus exposure) [34]. Additionally, some polyadenylation factors have been shown to be of great importance in the response of plants to environment changes, including Fip1 and CPSF30 (a core subunit of the CPSF complex). The extent of root inhibition by cadmium was significantly increased in the *fip1-2* compared to the wild-type *Arabidopsis* [29], and AtCPSF30 was shown to play a critical role in modifying the sensitivity of plants to oxidative stress [35] and in resistance to *Pseudomonas syringae* [36]. These findings suggest that polyadenylation promotes the adaptation of plants to their environment and that correct polyadenylation is crucial for plant development and stress responses.

## 3. Gene Expression Regulation in Plants via Coupled Transcription and RNA Processing

Nascent RNAs undergo several processing steps, including 5′ capping, splicing, 3′ polyadenylation, and chemical modification, to become mature mRNAs. Emerging evidence has shown interconnections among these processes, which appear to occur in series but may happen co-transcriptionally and even be coupled or interact with each other [37,38,39].

To date, two models have been proposed to explain these coupling processes in yeast and mammalian cells. In the first model, called the recruitment model, transcription is central to the coupling of different RNA processing events, mainly through RNA polymerase II (RNAPII). RNAPII serves as a platform for the recruitment of various processing factors to a nascent RNA, including capping factors, splicing factors, and polyadenylation factors [27]. For example, RNAPII that has been phosphorylated at serine 5 (Ser5P) in its carboxy-terminal domain (CTD) associates, specifically with the spliceosome during co-transcriptional splicing [40]. Besides RNAPII, chromatin itself can act as a platform for different processing factors; for instance, histone H3 trimethylated at lysine 36 (H3K36me3) can recruit RNA-binding proteins [41], and H3K4me3 can recruit the U2 small nuclear ribonucleoprotein (snRNP) to indirectly promote splicing [42].

In the second model, known as the kinetic model or kinetic competition model [43], the relative rates of transcription elongation and splicing or poly(A) site cleavage can affect the output of transcript isoforms and the relative content of different isoforms. The relatively slow elongation rate of RNAPII assists RNA processing factors by allowing more time for spliceosome assembly, the binding of processing factors, and recognition and cleavage at certain poly(A) sites [44]. More recently, it has been reported that the transcription elongation rate can also control RNA processing via changes in nascent RNA folding [45].

Current evidence suggests that these two models function in an interdependent fashion; the transcription elongation rate could influence the recruitment of different processing factors, and, in turn, the transcription elongation rate could be influenced by the differential recruitment of factors that control the elongation rate [37,46]. Studies in which the RNAPII elongation rate was altered suggest that a slower elongation rate allows more time for the recruitment of processing factors to a nascent RNA; however, this is not compatible with both the recruitment coupling and kinetic coupling models [47,48]. High-resolution analyses of transcription elongation rates and the nascent RNA structure are needed to understand the link between these two models. Moreover, it is unknown how these processes are coupled in plants and whether such coupling occurs similarly to that in mammals and yeasts.

### 3.1. Coupling of the Regulatory Events That Control Gene Expression in Plants

In addition to the coupling of transcription and RNA processing in mammals and yeasts, similar phenomena have been found in plants, particularly in studies of the model plant *Arabidopsis thaliana* (L.) Heynh. Here, we outline the current progress in understanding such coupling in plants, with a focus on polyadenylation.

#### 3.1.1. Coupling of Polyadenylation and Transcription in Delay of Germination 1 (DOG1) Expression

**Figure 1:**
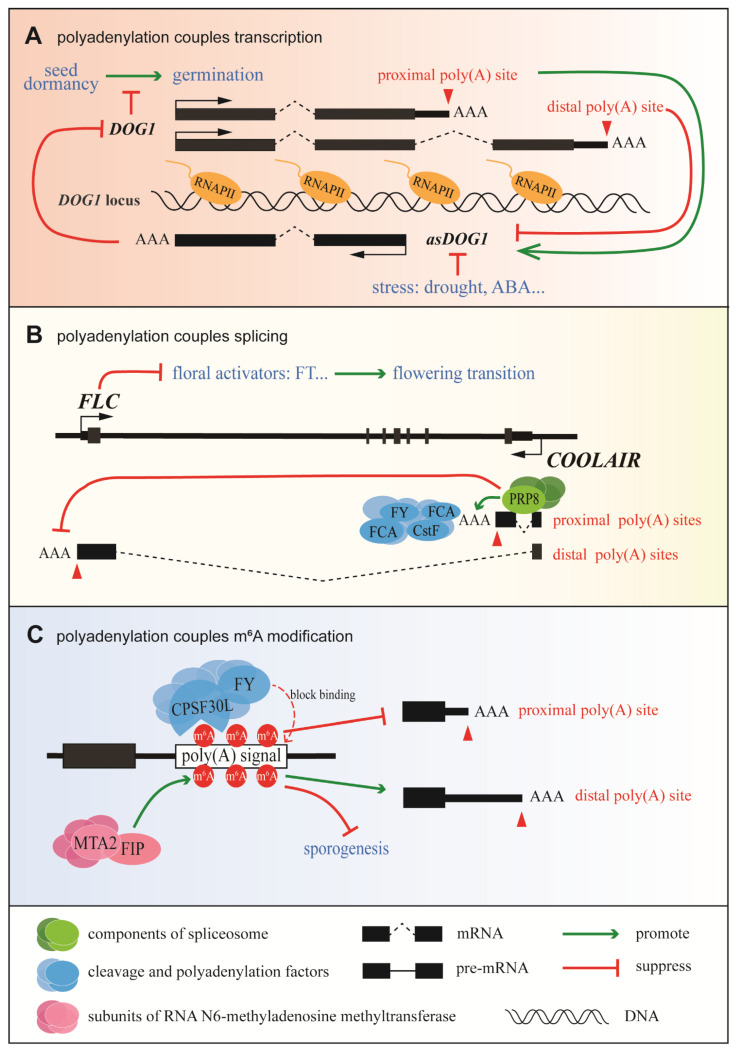
Schematic diagram of the coupling of transcription and RNA processing in plants. (**A**) Coupling between polyadenylation and the transcription of delay of germination 1 (*DOG1*) in *Arabidopsis*. Increasing usage of the *DOG1* proximal poly(A) site promotes the expression of *asDOG1*, which subsequently suppresses *DOG1* expression [55]. (**B**) Coupling between the polyadenylation and splicing of flowering locus C (*FLC*) transcripts in *Arabidopsis*. PRP8 facilitates the efficient splicing of the proximal intron in *COOLAIR* and promotes usage of the proximal poly(A) site in *COOLAIR* [31]. (**C**) Coupling between polyadenylation and m^6^A modification in plants. The m^6^A modification blocks binding of the polyadenylation factors CPSF30 and FY to the poly(A) signal, resulting in reduced usage of the proximal poly(A) site [56].

The coupling of polyadenylation and transcription in the control of *DOG1* expression is summarized in Figure 1A. Seed dormancy is important for plant survival; it is vital that seeds germinate at the right time [49]. *DOG1*, which was first identified as a major quantitative trait locus for seed dormancy in natural variations of different *Arabidopsis* accessions [50], is a key regulator of seed dormancy in *Arabidopsis thaliana* (L.) Heynh and other plants [51]. *DOG1* has two alternative poly(A) sites: polyadenylation of *DOG1* at the proximal site results in functional DOG1 (known as shDOG1), while polyadenylation at the less commonly used distal site results in the production of lgDOG1 [52]. 5′-RACE sequence data indicate that the 5′-capped antisense transcript known as *asDOG1* (or *1GOD*) [53] originates from the end of exon 2, which is close to the proximal poly(A) site of *DOG1*, and its promoter is located in the region of intron 2 and exon 3 [53]. *DOG1* is highly and specifically expressed in seeds, while it is nearly undetectable in seedlings [50,52]. However, *asDOG1* is mainly expressed in seedlings and has been shown to suppress *DOG1* expression in a cis manner during seed maturation [53]. Under normal conditions, *asDOG1* is expressed at a high level in seedlings and the expression of *DOG1* is suppressed to promote germination; however, when a stressor is applied, *asDOG1* is down-regulated and DOG1 accumulates to promote seed dormancy [54]. Thus, APA of sense transcripts controls the antisense transcription of *DOG1* in *Arabidopsis thaliana* (L.) Heynh [55], and *DOG1* affects the expression of *asDOG1* in *Arabidopsis thaliana* (L.) Heynh through APA, polyadenylation, and transcriptional co-regulation of seed dormancy (Figure 1A).

#### 3.1.2. Coupling between Polyadenylation and Splicing in Flowering Locus C (FLC) Expression

The most well-known model for the coupling of polyadenylation and splicing is the regulation of *Arabidopsis FLC* antisense transcripts (Figure 1B). *FLC*, which encodes a MADS-box transcription factor, plays a critical role in plant development as the primary inhibitor of flowering. *COOLAIR*, a collection of long antisense noncoding transcripts produced at the *FLC* locus, was first detected in 2007 [57]. Based on alternative splicing (AS) and APA, these transcripts can be separated into two classes: class I uses the proximal polyadenylation site located inside intron 6 of *FLC*, while class II uses the distal polyadenylation site within the *FLC* promoter. In addition to the use of APA sites, splice variants are produced that are closely related to different *FLC* expression states. For example, efficient splicing of the intron in class I transcripts promotes the usage of the proximal poly(A) site. This triggers the FLD-dependent demethylation of H3K4me2 in the *FLC* gene body, downstream of *COOLAIR*’s proximal poly(A) site, leading to the suppression of *FLC* expression and the promotion of flowering [31]. Initiation and elongation during *FLC* transcription are tightly coordinated, and both steps are affected by the status of the chromatin [58], which is thought to be controlled via a kinetic coupling mechanism. When the distal poly(A) site is used, the *FLC* expression level increases, resulting in late flowering. Adaptive studies of *Arabidopsis* accessions have shown that single, natural noncoding polymorphisms can significantly change the splicing pattern of class II transcripts [59], resulting in an altered secondary structure of *COOLAIR* [60] and the up-regulation of *FLC* expression. Taken together, these results suggest that a precise regulatory network exists during *FLC* transcription to couple polyadenylation and splicing.

#### 3.1.3. Coupling between Polyadenylation and m^6^A Modification in Plants

Recent data suggest that m^6^A functions as a stabilizing factor by suppressing local ribonucleolytic cleavage [61]. Other data show that m^6^A can suppress the use of proximal poly(A) sites and that adenosines within the AAUAAA signal may be methylated [57] (Figure 1C). CPSF30 (the 30 kDa subunit of CPSF) and WDR33 in mammals are responsible for the recognition of AAUAAA via direct sequence binding [16]. Furthermore, CPSF30L, a homologous isoform of CPSF30, has a YTH domain, enabling it to bind RNAs specifically at the m^6^A position [17], while flowering locus Y (FLY), a homologue of human WDR33 [16], is associated with the recognition of poly(A) signals in plants [62]. The m^6^A modification may either directly block the binding of CPSF30 and FLY to poly(A) signals or the binding of m^6^A by YTH domain-containing proteins, thereby inhibiting the recognition of poly(A) signals by CPSF30 and FLY [50]. In addition, FKBP12-interacting protein (OsFIP) and mRNA adenosine methylase 2 (OsMTA2), two subunits of the RNA m^6^A methyltransferase complex, which function in sporogenesis, can bind to mRNAs-encoding threonine proteases and NTPases at the early microspore stage, mediating their m^6^A modification and affecting their expression and/or splicing [63] (Figure 1C). Meanwhile, a transcriptome-wide analysis revealed that m^6^A modification is highly coordinated with APA site usage [64]. Overall, despite the fact that little is known about m^6^A modification in plants, evidence suggests that it plays a crucial regulatory role in the coupling of transcription with RNA processing.

In summary, though the regulation of polyadenylation in plants is poorly understood, the above findings confirm that coupling between polyadenylation and transcription, splicing, or m^6^A modification exists in plants.

### 3.2. Critical Proteins Involved in the Coupling of Polyadenylation and Other RNA Processing Events

The above-mentioned studies showing the existence of coupling between polyadenylation and other RNA processes in plants add several regulatory layers to gene expression that may help fine-tune the response to stressful conditions or provide a benefit during development. Still, important questions remain. For example, how are these seemingly distinct processes integrated via the collaboration of different processing factors? When and where does this regulation occur? In the next section, we provide a summary of proteins that may participate in the coupling of regulatory processes during gene expression in plants.

#### 3.2.1. RNAPII

RNAPII is the best-known regulatory protein involved in this type of coupling; it has been studied in plants, mammals, and fungi. In eukaryotes, RNAPII plays a fundamental role in gene expression. RNAPII and various phosphorylated CTD isoforms are involved in different stages of the transcription cycle [65,66]. RNAPII interacts with many cleavage and polyadenylation factors, including polyadenylation factor protein 1 of cleavage factor 1 (Pcf11), a component of the CFII cleavage and polyadenylation core complex [67], and Yhh1p/Cft1p, a yeast homologue of human CPSF160, which binds specifically to the phosphorylated RNAPII CTD [68].

A genome-wide analyses of *Arabidopsis* demonstrated that RNAPII with an unphosphorylated CTD mainly gathers downstream of the transcription start site (TSS), while RNAPII with a Ser5P CTD is required for co-transcriptional splicing; 5′ SS cleavage was achieved through an interaction between the spliceosome complex and Ser5P CTD during the elongation phase of transcription. In addition, RNAPII with a Ser2P CTD paused immediately downstream of the polyadenylation site [69]. Pcf11p-similar protein (PCFS4), a homologue of yeast Pcf11p in *Arabidopsis thaliana* (L.) Heynh, possesses a C-terminal interaction domain (CID), which is responsible for its interaction with the RNAPII CTD, while its C-terminal region mediates interactions with the polyadenylation factor Clp1-similar protein 3 (CLPS3) [27,67]. RNAPII may thus act in plants as a platform to recruit polyadenylation factors like PCFS4 to promote the cleavage and polyadenylation of a nascent RNA as soon as it is synthetized.

#### 3.2.2. U1 snRNP

Among the proteins that are critical in coupling RNA transcription and processing is U1 snRNP, the most abundant snRNP in most eukaryotes [70]. U1 snRNP is known for its vital role in the initial recognition of 5′ splice sites (SSs) [71]. Purified human U1 snRNP consists of U1 snRNA, Sm proteins, and three U1-specific proteins: U1A, U1C, and U1-70K [72]. Among these subunits, U1A and U1-70K contain a polyA polymerase (PAP) inhibition motif, which interacts with PAP to inhibit polyadenylation [73]. Early studies indicated that U1 snRNP inhibits cleavage and polyadenylation in different ways according to the position of its binding site relative to the poly(A) site. When U1 snRNP binds to the 5′ SS downstream of the proximal poly(A) site, it inhibits cleavage but does not affect the recruitment of other cleavage and polyadenylation factors. In contrast, when U1 snRNP binds to the terminal exon upstream of the PAS, it inhibits polyadenylation [74]. Subsequent genome-wide studies found that the role of U1 snRNP is not carried out in a gene-specific manner [70]. U1 snRNP usually binds to nascent RNAs through base pairing with the U1 snRNA’s 5′ sequence to suppress actionable PASs located in introns, and the binding is associated with RNAPII [66]. U1 snRNP participates in a complex with cleavage and polyadenylation factors (U1-CPAFs) that is distinct from U1–spliceosome complexes; it regulates 3′-end processing and the elongation and termination steps of transcription [75]. In addition, the impact of U1 snRNP is dose dependent [76].

Most protein components of yeast U1 snRNP are conserved in plants [77,78] (Table 2), suggesting a similar function for U1 snRNP in plants. Recently, *Arabidopsis* U1 (AtU1)A was found to be closely associated with AtU1-70K and AtU1C, confirming its essential role in pre-mRNA splicing in *Arabidopsis thaliana* (L.) Heynh [79].

#### 3.2.3. U2 snRNP

Previous studies also found that the interaction between CPSF and U2 snRNP contributes to the coupling of splicing and 3′-end formation in mammals and fungi [83,84]. U2 snRNP binds the intron’s branch site near the 3′ SS through base pairing of the U2 snRNA with the branch site [85]. U2 snRNP auxiliary factor 65 (U2AF65), a splicing factor that promotes pre-spliceosome assembly [86], interacts directly with the 59 kDa subunit of CFIm (CFIm59), and CFIm59/25 heterodimers promote cleavage and polyadenylation [84]. U2 snRNP also interacts with subunits of CPSF directly through its component SF3b [83]. The physical and genetic interactions between the spliceosomal RNA helicase Prp5p and Spt8p/Spt3p, components of the Spt–Ada–Gcn5 acetyltransferase complex, balance transcription initiation/elongation and pre-spliceosome assembly/proofreading in yeast [87].

There are four *U2AF65* homologues predicted in the *Arabidopsis* genome, but only two of them have a U2AF homology motif [80] (Table 2). U2AF65 interacts with *Arabidopsis* splicing factor 1. Intriguingly, plant SF1 homologues have an additional RNA recognition motif (RRM), which is not present in their yeast and mammalian counterparts [88] and which may be critical in the recognition of nascent RNAs.

#### 3.2.4. Suppressor of Ty 5 (Spt5)

Another critical protein in the control of gene expression via coupled RNA production/processing is the transcription elongation factor Spt5, which is conserved in all domains of life [89]. The C-terminal repeat region (CTR) of Spt5 resembles the RNAPII CTD, which is an intrinsically unstructured extension [90]. The Spt5 CTR sequence varies across species; however, they all contain residues that can be phosphorylated [91]. Dynamic phosphorylation of the Spt5 CTR acts as a switch to promote or suppress transcription elongation [92]. In addition, DRB sensitivity-inducing factor, formed from Spt5 and Spt4, regulates the processivity of RNAPII during elongation through direct interaction with the polymerase [93] and is involved in efficient termination of transcription [94]. When the transcription complex passes over the PAS, Spt5 is dephosphorylated and the Ser2P form of the RNAPII CTD accumulates [95], causing RNAPII to decelerate and become a viable target for the nuclear exonuclease Xrn2 [92]. This is in line with the binding peak of Spt5 discovered downstream of poly(A) sites [94]. Additionally, a physical interaction was detected in yeast between Spt5 and pre-mRNA processing protein 40 (Prp40) [96], which associates with U1 snRNP in the early steps of spliceosome assembly [97]. Further, interactions have been observed between Spt5 and all five spliceosomal snRNAs (U1, U2, U4, U5, and U6). Spts affect the recruitment of U5 snRNP to intron-containing genes [98]. Remarkably, the flexible nature of its CTR enables Spt5 to act as a landing platform for different protein factors, including transcription elongation factors [99], splicing factors, and 3′ cleavage and polyadenylation factors [100]. The first study of Spt5 in plants was published in 2014; thus, research into its function in plants is ongoing [101]. Nevertheless, studies have shown that the Spt4/Spt5 heterodimer is conserved in plants, and Spt5 both co-localizes with RNAPII during transcription [101] and interacts with vernalization independence 5 (VIP5), a core component of the *Arabidopsis* RNA polymerase II-associated factor 1 complex, through its phosphorylated CTR [81] (Table 2). Therefore, the conserved CTR in *Arabidopsis* Spt5 may have similar functions to that in yeast and mammals and may be essential for the coupling of multidimensional processes.

#### 3.2.5. The THO/TREX complex

In human and yeast cells, mRNA synthesis and processing are coupled to nuclear mRNA export, during which the TRanscription-EXport (TREX) complex plays an extraordinary role. The TREX complex is composed of the THO complex and a group of additional proteins that are conserved across species, including *Saccharomyces cerevisiae*, *Arabidopsis thaliana* (L.) Heynh, *Drosophila*, and humans [102]. In yeast, the TREX complex is recruited to RNAPII co-transcriptionally through the direct interaction of its subcomplex (THO) with the Ser2-phosphorylated CTD of RNAPII [103]. Intriguingly, the pre-mRNA processing factor 19 splicing complex, also called the NineTeen complex, was found to facilitate the recruitment of the TREX complex to transcribed genes as well [104]. Moreover, the RNA export adaptor yeast RNA annealing protein 1 (Yra1), a subunit of the TREX complex, competes with cleavage factor polyribonucleotide kinase subunit 1 (Clp1) to interact with the 3′-end processing factor Pcf11, and the dynamic balance between Pcf11-Yra1 and Pcf11-Clp1 complexes affects the final selection among different poly(A) sites [105]. Meanwhile, Pcf11 plays a central role in coupling 3′-end processing with transcription via a direct interaction between its CID and the CTD of RNAPII [67]. Thus, the TREX complex, together with these various factors, links transcription, RNA processing, and nuclear export.

Notably, the *Arabidopsis* THO core complex consists of hyper recombination 1 (HPR1), TEX1 (THO3), THO2, THO5A/B, THO6, and THO7A/B [82] (Table 2). HPR1 and TEX1 are the best studied of these components. Several reports have shown abnormal splicing patterns in *hpr1*, *tex1*, and *tho2* plants [106,107]. In addition, several splicing factors have been co-purified with TEX1, and TEX1 was found to co-localize with the splicing factor SEINE-ARGININE-RICH (SR) protein family members RSZ22 and RSZ33 [108]. HPR1 was also found to co-localize with the SR protein family member SR33 in the nucleus [106], indicating co-localization between the *Arabidopsis* THO/TREX complex and splicing factors, consistent with observations in human and yeast cells [104]. More recently, aberrant 3′-UTR extensions were discovered in *tex1* and *hpr1*, the majority of which were shared, suggesting their roles in polyadenylation [109]. PCFS4, the *Arabidopsis* ortholog of yeast Pcf11, possesses a CID as well, which can interact with the RNAPII CTD [67]. CLPS3, an ortholog of yeast Clp1, is also conserved in *Arabidopsis thaliana* (L.) Heynh [27]. However, little is known about their roles associated with the TREX complex; instead, plant studies of the THO/TREX complex have focused on the biogenesis of small RNAs, including microRNAs, small interfering RNAs (siRNAs), and *trans*-acting siRNAs [107,110,111].

### 3.3. LLPS Enables Efficient Coupling of Transcription and RNA Processing in Plants

In addition to the proteins mentioned above, specific cytosol condensates may provide appropriate spatial environment for the coupling of transcription and RNA processing.

#### 3.3.1. Phase-Separated Membraneless Organelles

Membraneless organelles formed through liquid–liquid phase separation (LLPS) may play a general role in the machinery that is responsible for co-transcriptional mRNA processing; they could provide spatial possibilities for diverse biochemical reactions to take place efficiently at the same time without disturbing each other or perturbing the intracellular environment. Over the past decade, phase separation has become an emerging field, with studies aimed at determining how cells organize diverse functions while maintaining cellular homeostasis [112].

LLPS is a subtype of phase separation that is known to be sensitive to environmental changes; it can respond rapidly to intracellular fluctuations because of its reversible and dynamic nature [113]. Phase separation is driven by interactions among molecules with multivalent domains or intrinsically disordered regions (IDRs) [114]. The proteins involved in LLPS exhibit low sequence complexity and no defined tertiary structure and can alternate between multiple conformations rapidly [115]. The highly flexible structure of intrinsically disordered proteins (IDPs) is of great importance in the establishment and maintenance of nuclear compartments [116]. IDPs are essential for optimal enzyme activity [117], function as hubs in signaling networks [118], act in metabolic regulation [119], and enable stress responses [120,121] through interactions with various partners in membraneless compartments [122].

Various functions in plants rely on proteins with IDRs, including DELLA, CRY, BKI1, BAK1, and ELF3 [121,122,123]. A specific nuclear LLPS condensate of the polyadenylation complex has been observed in *Arabidopsis thaliana* (L.) Heynh. The RNA-binding protein flowering control locus A (FCA) associates with a coiled-coil protein, FLX-like 2 (FLL2), to promote the formation of liquid-like bodies, which concentrate near 3′-end processing factors at specific poly(A) sites. Both FCA and FLL2 are IDPs with disordered domains [124]. IDPs are widespread in eukaryotes; a genome-wide analyses indicated that the *Arabidopsis* proteome consists of approximately 30% IDPs [125]. These IDPs can lead to spontaneous nucleation, form phase-separated condensates that resemble non-membrane-bound organelles, and separate from the surrounding phase [126]. A concentrated reaction zone can thus be created, allowing for more efficient biochemical interactions among IDPs and their partners. As a result, different types of regulation (including the coupling of transcription with RNA processing) can take place in distinct spaces in the crowded intracellular environment.

#### 3.3.2. The Underlying Coupling Regulation Model

It is worth stressing that the membraneless compartments and important proteins mentioned above are not isolated from each other, but interrelated. For instance, Spt5 interacts physically with Prp40, a core protein in the U1 snRNP [96]. Additional evidence has been provided by proteomic analyses; numerous interactions among the transcription elongation complex, splicing factors, and cleavage and polyadenylation factors were found in *Arabidopsis thaliana* (L.) Heynh through co-purification with RNAPII and transcription elongation factors [127]. The RNAPII CTD, which is conserved across eukaryotes, contains tandem heptapeptide repeats that form a mobile extension from the catalytic core of RNAPII [128]. This IDR of the RNAPII CTD confers physicochemical properties beyond those predicted by its sequence that can mediate multivalent interactions during LLPS [129].

**Figure 2:**
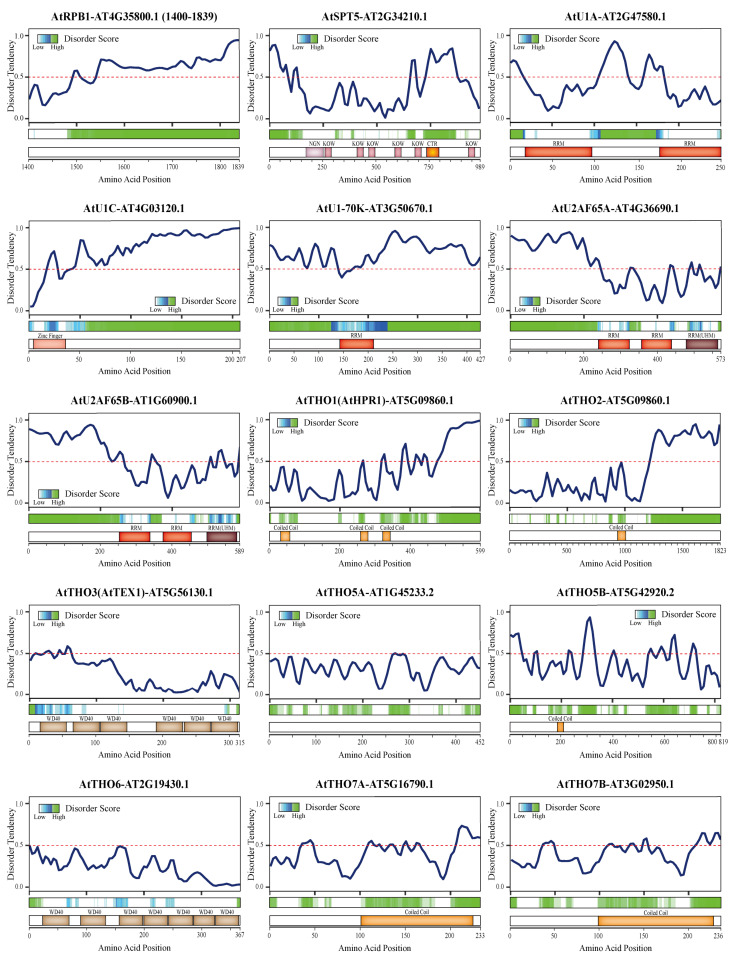
Disorder tendency predictions for proteins involved in the coupling of transcription and RNA processing in plants. All protein sequences were downloaded from TAIR (https://www.arabidopsis.org/ (accessed on 6 November 2020)). (For each figure, **top panel**) Protein disorder tendency curve. The disorder tendency score of each amino acid was predicted using IUPred2A [134] and subsequently fit to a smooth curve using the R ggplot2 package. The predicted scores were between 0 and 1, with a score above 0.5 (dashed line) indicating disorder. (For each figure, **middle panel**) Prediction of disordered regions using D^2^P^2^ [135]. (For each figure, **bottom panel**) A domain map of the proteins in *Arabidopsis* based on previous studies [81,88] and the protein databases UniPro [136], Pfam [137], and SMART [138]. The disorder predictions for full-length proteins, except for AtRPB1 (C-terminal domain), are displayed. Abbreviations: WD40, tryptophan-aspartic acid motif repeats; RRM, RNA recognition motif; UHM, U2AF homology motif; NGN, NusG N-terminal domain; KOW, Kyrpides–Ouzounis–Woese motif; CTR, C-terminal repeat region.

We used a variety of tools to predict the disorder tendency of the other proteins mentioned above, with a focus on proteins from *Arabidopsis thaliana* (L.) Heynh (Figure 2). Interestingly, all of the proteins and some protein complex subunits were predicted to have highly disordered regions, suggesting that they have the ability to form or promote LLPS. Consistent with our predictions, U1-70K was reported to undergo LLPS via its low-complexity regions [130]. In addition, a few reports have suggested specific roles for the chemical modifications involved in phase separation. For example, H3K9me3 governs heterochromatin formation via phase separation [131], while the number and distribution of m^6^A modifications affect the phase separation potential of mRNAs [132]. Additionally, phosphorylation of the RNAPII CTD oversees the partition of RNAPII into phase-separated condensates, thereby ensuring that RNAPII functions normally in transcription and RNA processing [133]. Strikingly, the CTR of Spt5, which is a low-complexity region, can be phosphorylated by cyclin-dependent kinase 9, which also controls the phosphorylation state of the CTD of RNAPII [95]. Apart from these reports of proteins (e.g., the RNAPII CTD and U1-70K) and chemical modifications that can undergo or promote LLPS, additional proteins and protein complexes have the potential to participate in LLPS based on our disorder prediction results (Figure 2). Some of them, like the RNAPII CTD, may act as a scaffold or driver of the condensate, while others may participate as client proteins that are mobile depending on the detailed functional requirements, and still others may play different roles in distinct condensates. These membraneless biomolecular condensations can be quite beneficial for achieving efficient regulation in a crowded environment.

**Figure 3:**
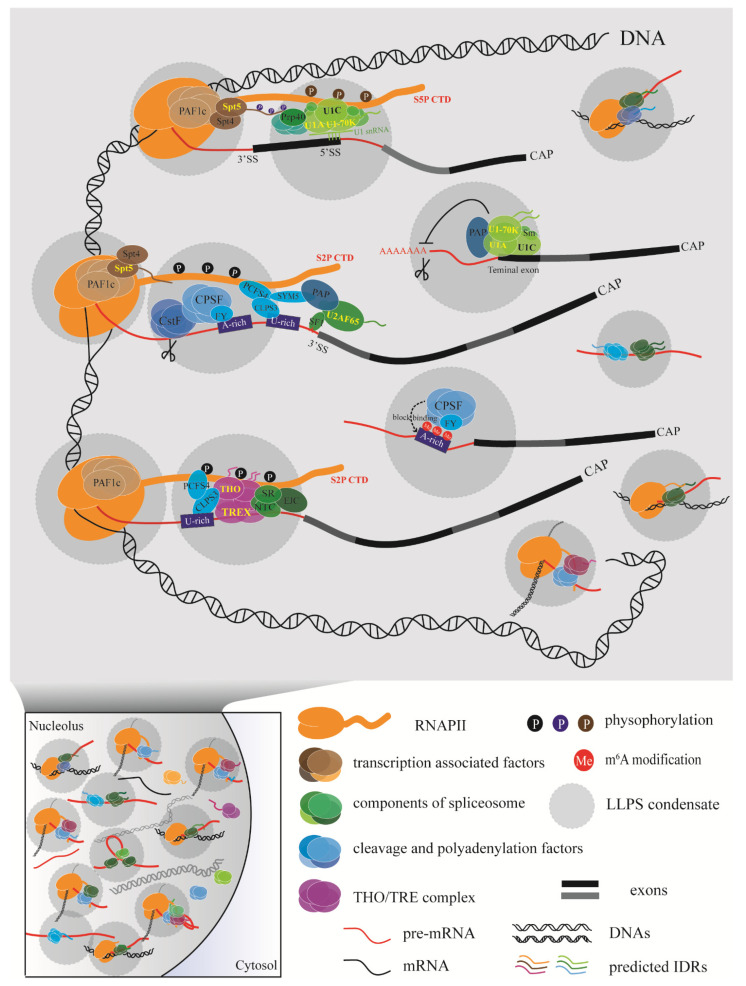
Schematic diagram of the coupling machinery generated through liquid–liquid phase separation (LLPS) in plants using different processing factors. The various regulatory processes are carried out efficiently inside the phase-separated condensate, which may be driven by the carboxy-terminal domain (CTD) of RNAPII or RNA processing factors with intrinsically disordered regions (IDRs).

A model of the events involved in coupling transcription with RNA processing has emerged (Figure 3). In this model, diverse factors interact with each other at the right time according to the requirements for transcription and RNA processing. A relatively slow RNAPII elongation rate leaves much more time for polyadenylation factors to assemble properly. Conversely, a fast RNAPII elongation rate may lead to improper polyadenylation, affecting the recognition of a weak poly(A) site or modification site and resulting in chaotic consequences for the cell. With so many processing factors around RNAPII and the nascent RNA, how do the diverse biochemical reactions proceed in order without disturbing each other? Multivalent regulation could allow reactions to occur efficiently inside phase-separated condensates formed by the RNAPII CTD or RNA processing factors with IDRs.

## 4. Conclusions and Perspectives

Once a primary RNA transcript has been synthesized, it must be processed before becoming a fully functional mRNA. Processing includes 5′ capping, splicing within the transcript body, 3′ polyadenylation, and (sometimes) chemical modification. These complex events cannot be easily separated from each other in time and space; however, partitioned membraneless organelles formed by LLPS may greatly improve the accuracy and efficiency of this process.

One interesting question is how plants achieve cleavage and polyadenylation at a precise position using conserved core factors when they lack the conserved PAS sequences found in animals and yeast. One possibility is that plant-specific proteins, such as plant-specific RBPs, are involved in the regulation of polyadenylation in plants. RBPs are of great importance in RNA–protein interactions, which are characterized by the recognition of a specific sequence or structural element in the target RNA by an RNA-binding domain (RBD) such as an RRM or K homology domain [139]. In addition, RBPs appear frequently in LLPS [140]; the sequence-binding specificity of different RBDs may determine the processing of a certain subset of nascent RNAs in a particular droplet. High-throughput studies have shown that the RNA-binding proteomes of plants can be divided into three categories: RBPs containing classical RBDs (22%), RBPs containing non-classical RBDs (39%), and RBPs containing unknown RBDs (39%) [141]. RBPs lacking known RBDs are suspected to be plant specific, and several plant-specific RBPs have been reported to function in plant-specific biological processes such as flowering and photosynthesis (reviewed in [142]). Together, these results provide direction for further study of polyadenylation in plants, with an emphasis on the importance of plant-specific RBPs.

## Figures and Tables

**Table 1:** Homologues of core cleavage and polyadenylation factors in plants.

Complex	Subunits	*Arabidopsis*	References	Recognize Sequence	References
Cleavage and polyadenylation specificity factor (CPSF)	CPSF160 (CPSF1)	AtCPSF160	[15]	AAUAAA	[16,17]
	CPSF100 (CPSF2)	AtCPSF100	[18]		
	CPSF73 (CPSF3)	AtCPSF73-II	[15]		
	CPSF30 (CPSF4)	AtCPSF30	[19]		
	WDR33	FY	[20]		
	Fip1	AtFIP1	[21]		
Cleavage stimulation factor (CstF)	CstF50 (CstF1)	\	\	U/G-rich region	[22]
	CstF64 (CstF2)	AtCstF64	[23]		
	CstF77 (CstF3)	AtCstF77	[23]		
Cleavage factor I (CFI)	CFIm25	\	\	UGUA	[24]
	CFIm68	\	\		
	(CFIm59)	\	\		
Cleavage factor II (CFII)	CLP1	Clp1-similar protein 3 (CLPS3)	[25]	G-rich region	[12,26]
	PCF11	Pcf11p-similar protein (PCFS4)	[27]		

**Table 2:** Homologues of critical proteins involved in the coupling regulation in plants.

Proteins	*Arabidopsis*	References
U1A	AtU1A	[77]
U1C	AtU1C	
U1-70K	AtU1-70K	
U2AF65	AtU2AF65a	[80]
	AtU2AF65b	
Spt5	SPT5	[81]
THOC1 (Hpr1)	HPR1	[82]
THOC2	THO2	
THOC3 (TEX1)	THO3 (TEX1)	
THOC5	THO5A	
	THO5B	
THOC6	THO6	
THOC7	THO7A	
	THO7B	
